# Simulation-driven design of smart gloves for gesture recognition

**DOI:** 10.1038/s41598-024-65069-2

**Published:** 2024-06-27

**Authors:** Clayton Leite, Petr Byvshev, Henry Mauranen, Yu Xiao

**Affiliations:** Department of Information and Communications Engineering, Aalto University, Konemiehentie 2, 02150 Espoo, Finland

**Keywords:** Smart-glove design, Sensor placement optimization, Simulation-driven design, Sensor simulation, And data augmentation, Computer science, Software

## Abstract

Smart gloves are in high demand for entertainment, manufacturing, and rehabilitation. However, designing smart gloves has been complex and costly due to trial and error. We propose an open simulation platform for designing smart gloves, including optimal sensor placement and deep learning models for gesture recognition, with reduced costs and manual effort. Our pipeline starts with 3D hand pose extraction from videos and extends to the refinement and conversion of the poses into hand joint angles based on inverse kinematics, the sensor placement optimization based on hand joint analysis, and the training of deep learning models using simulated sensor data. In comparison to the existing platforms that always require precise motion data as input, our platform takes monocular videos, which can be captured with widely available smartphones or web cameras, as input and integrates novel approaches to minimize the impact of the errors induced by imprecise motion extraction from videos. Moreover, our platform enables more efficient sensor placement selection. We demonstrate how the pipeline works and how it delivers a sensible design for smart gloves in a real-life case study. We also evaluate the performance of each building block and its impact on the reliability of the generated design.

## Introduction

**Figure 1:**
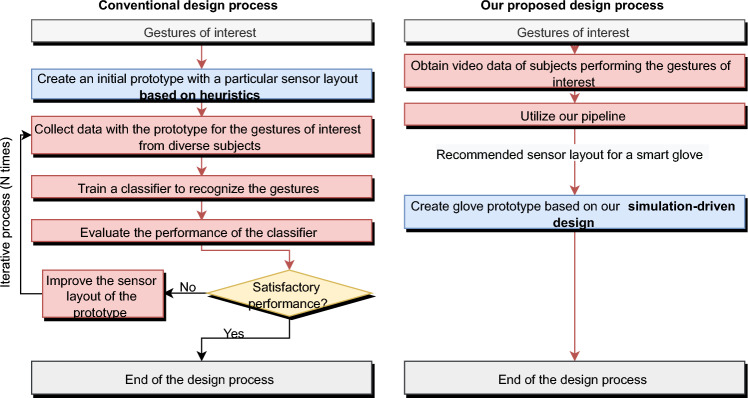
A comparison between the traditional design process of smart gloves and our proposed solution. In each iteration of the conventional design process, time and labor costs are involved in modifying the prototype, collecting data once more, training a new classifier with the newly obtained data, and evaluating the modified prototype. The output of the design process is the sensor placement and a trained classifier.

Smart gloves are emerging human–computer interfaces for augmented and virtual reality applications^1^. They support finger and hand tracking, as well as gesture and activity recognition, by utilizing various sensors (e.g., accelerometers, gyroscopes, pressure/flex sensors, and magnetic field sensors) embedded throughout the gloves. Currently, designing a smart glove for a specific application is usually a complex and costly process that involves trial and error tests. These tests aim to optimize the selection of sensor modalities and their locations (i.e., sensor placement optimization), refine the sensor data collection protocols, and set the training and evaluation of data processing models. The iterative design process involves intensive labor work for creating glove prototypes and conducting user studies, as illustrated in Fig. 1. Organizing user studies is particularly time-consuming, necessitating the preparation of physical spaces, participants, and equipment to collect accurate ground truth data.

Simulation-driven design has been proposed to reduce the manual effort required for data collection and prototyping by simulating sensor data, which can be used for training classification models^2–5^. In this paper, we propose an open platform that implements a pipeline for simulation-driven smart glove design. By using video or motion data as input, our platform provides optimized sensor layouts and classification models as output. Compared to existing simulation-driven platforms^2–5^, our platform addresses two key challenges that relate to the input and optimization of sensor placement.

Existing platforms always require precise motion data (i.e., 3D human poses) as input. Via a process called inverse kinematics^6,7^, the input is transferred to a human model in a virtual environment, which allows it to be animated exactly as the gestures and activities of interest. Subsequently, these platforms generate artificial readings for virtual sensors placed at arbitrary locations on the human model, a step known as “sensor simulation.” The simulated sensor data is then used to train classifiers. Any errors in the input data can negatively impact the subsequent steps, leading to unreliable designs. Obtaining highly accurate motion data requires complex and expensive camera systems. As an alternative, monocular video data are easy to obtain due to the widespread availability of smartphones and webcams. However, estimating 3D human poses from monocular videos often results in erroneous data.

Our platform supports monocular videos as input and minimizes the impact of errors through a two-step process. Firstly, several pre-processing transformations to the 3D pose estimations (which were extracted from monocular videos) are applied before passing them through an inverse kinematics module. These transformations, which include scaling, translating, and rotating each estimated pose, aim to correct errors from inaccurate scale, position, and orientation estimations. Second, the results of the inverse kinematics are employed to identify and filter out the worst pose estimations. To compensate for the reduced dataset size due to filtering, we augment the dataset by varying hand size and motion dynamics.

In addition to sensor simulations based on motion data, sensor placement optimization is another key component of a simulation-driven platform. Instead of conducting exhaustive search to find the optimal placement, which is extremely time-consuming, previous researchers^3,8–12^ have formulated sensor placement as an optimization problem focused on maximizing classification performance under the constraints of the number of sensors. For instance, Xia and Sugiura^3^ employed the Cuckoo Search algorithm, a heuristic approach that explores possible sensor placements by training a classifier on artificial sensor readings and updating the placement to maximize performance. In this paper, we share the same objective but achieve it by filtering out irrelevant or redundant locations based on hand joint independence and correlation before training classifiers. Our approach is more intuitive, as it relies on hand joint angles rather than non-human-readable data such as accelerations, angular speeds, and magnetic readings.

We built our platform on top of open source software, including OpenSim^6^ and MediaPipe^13^, and provided a thorough evaluation of its capabilities. In detail, we used open video and hand motion datasets to evaluate the quality of the video-based hand motion extraction, which is reflected in the precision of the inverse kinematics fit (the average error for hand joint angles is examined). We reported the accuracy of sensor simulations by comparing the simulated sensor readings derived from motion capture data with the ground truth sensor records for a typical commercial IMU sensor (*Movesense*). We trained the model with data augmented through specialized techniques for motions, such as hand size and gesture style augmentations, and demonstrated the superior performance of the style augmentation. Finally, we developed a case study that involved running the entire pipeline to design a smart glove for a set of common gestures. In this case study, various prototypes were built and their gesture recognition performance was evaluated. The best-performing prototype aligned with the smart glove design proposed by our pipeline.

The scientific contributions of our work are summarized in the following list.

1. To address the unreliable and inaccurate input data, we devise various techniques: (1) a series of preprocessing transformations on the 3D pose estimations extracted from video data, (2) detection and filtering out of the worst estimations based on the results of inverse kinematics, and (3) counteracting the filtering by enriching the dataset with augmentation techniques.
2. We developed a sensor placement optimization algorithm that analyzes the statistics of finger joint angles for gestures to derive a potential optimal configuration of sensors. In comparison to other sensor placement solutions^3,8–12^, our method works on finger joint angles, which are more tractable and intuitive to analyze than three-dimensional accelerations, angular speeds, and magnetic readings. This also results in a less computationally demanding algorithm. Additionally, by using heuristics based on finger movements, our sensor placement optimization algorithm requires fewer data to generate an optimized design.

Our pipeline and the presented insights can be useful for engineers and practitioners who want to design a smart glove for a specific small-scale use case since it allows them to do so without having to resort to complex and expensive data collection.

The rest of the paper is organized as follows. Section “Background and related work” covers the background and related work, focusing on design through simulation and gesture recognition. Section “Methods” provides a detailed description of the proposed simulation pipeline. Section “Experimental results” presents experiments on the proposed pipeline and reports the performance of each step. Section “Discussion” discusses the benefits, limitations, and future work related to the proposed technology. Finally, the paper concludes in Sect. “Conclusions”.

## Background and related work

In this section, the background technologies used for building the pipeline are presented, including vision-based motion extraction and IMU sensor simulation. Additionally, previous works related to simulation-driven designs for wearable devices are discussed.

### Sources of human motion data

Human motion data are typically collected from infrared cameras and reflective markers, which are then linked to a skeletal estimate of the human body. Until recently, methods based on any other sensor technology have been too imprecise for this purpose. The current state-of-the-art in human motion tracking solutions, with the highest reported accuracy, is achieved by employing infrared camera-based motion capture systems such as OptiTrack, VICON, and VisualEyez. These systems claim to achieve precision levels ranging from 76 µm to 1 mm in tracking^14,15^. However, they are prohibitively expensive.

Visual data such as photos and videos can be used to extract information about human body mechanics, such as body poses, motion dynamics, and various interactions with the surrounding environment^16–18^. OpenPose and MediaPipe are two major tools that are used to extract body key-point information in many applications. Such key-points are usually associated with major human body joints and facial features (i.e., the eyes, mouth, shoulders, elbows, hands, hips, knees, ankles, etc.). OpenPose^19^ was one of the first systems to detect multiple body and hand key-points from raw video frames. However, it is limited in its processing speed and accuracy, which made it ill-suited to our purposes. MediaPipe^13^ is a framework that can be used to build perceptual models. It integrates various machine learning solutions such as face detection, hand, and finger tracking, and body pose tracking. In this work, MediaPipe is utilized to extract 3D hand pose information from monocular videos since it has been proven to be faster and more accurate than OpenPose^20,21^.

Another option for collecting motion data is synthesizing new motions from existing ones. Several different approaches follow this direction. Ma et al.^22^ and Zhou et al.^23^ generated variants of example motions in different styles by using Gaussian processes and key pose extraction. Kulić et al.^24^ generated new combinations of movement from extracted motion primitives by using hidden Markov models. More modern approaches use deep reinforcement learning to move a kinematic skeleton, which Luo et al.^25^ and Zhang et al.^26^ did for quadrupeds and which Lee et al.^27^ did for human bodies. These methods were developed with mostly animations in mind and require a control signal (e.g., direction changes during walking) and were, therefore, not suitable for our purposes without significant changes. Creating a deep reinforcement learning model for our needs would require large quantities of real-world measurements and significant work to adjust the models. Hence, we built our augmentation methods based on Ma et al.’s^22^ work.

### IMU sensor simulation

As mentioned in Sect. “Introduction”, the sensor simulation is a key component in a simulation-driven pipeline. It aims to generate artificial sensor readings from the motion data. Here, several studies on sensor simulation are introduced, focusing on IMU sensors, as they are the most common type of sensors.

Young et al.^28^ designed IMUSim, which is an open-source simulation environment that generates artificial IMU sensor readings from continuous trajectory models of human motion. IMUSim simulates a comprehensive set of real-world issues that are seen in IMUs such as noise, bias, axis misalignment, and cross-axis sensitivity. Magnetic field distortions and network-related issues such as radio packet loss are also considered in IMUSim. In various experiments, the authors have demonstrated a good correspondence between the generated and real IMU sensor readings. Similarly, Brunner et al.^29^ developed an in-depth realistic model for simulating IMU readings from trajectory data.

In MEASURed^30^, accelerometer readings are generated from motion capture data by simply obtaining the second derivative of the position regarding time. Despite considerable differences between the generated and the real accelerometer data that are caused by the simplistic simulation approach, experiments demonstrated that the performance of activity classifiers that were trained on synthetic data was similar to the performance of those that were trained on real accelerometer data. Therefore, the authors demonstrated that–given the high-quality trajectory data from human motion—it is possible to estimate in a simulated environment how a certain configuration of accelerometers (including their quantity, placement, and sampling frequency) will perform in real life. Similar studies on error-free IMU simulation were published by Mundt et al.^31^ and Kang et al.^4^.

The previously mentioned methods for simulating IMU data were either discontinued, lack proper documentation, such as IMUSim^28^, which is unavailable for use (closed source code), or do not employ a realistic mathematical model. However, The MATLAB^32^ software—used in our pipeline for IMU simulation—is easily accessible and simple to use. It accommodates a family of functions for realistic IMU simulation that cover a comprehensive set of error signals from systematic errors such as the effects of the environmental temperature on the measurements of the IMU and the influence of the analog-to-digital conversion to non-systematic ones. MATLAB also supports the simulation of IMU data that are combined with GPS measurements for navigation applications. The complete mathematical model of MATLAB’s IMU sensor simulation is available on the software’s documentation page^33^. Such a mathematical model can be easily implemented in a Python script based on the documentation provided by MATLAB. Hence, even if the user is not able to access the MATLAB software package, which is not free, it is still possible to make use of MATLAB’s sensor simulation.

### Simulation-driven design

A simulation-driven design is characterized by a virtual environment where different versions of a design can be easily simulated and evaluated. This allows users to select the best-performing design. Asare et al.^5^ proposed a virtual experimentation platform that they named BodySim. BodySim contains a human structure model that mimics human motions that have been recorded with motion capture systems. BodySim also supports the installation of virtual IMU sensors in diverse locations on a human model; the readings are simulated on IMUSim^28^. Thus, BodySim can be used to develop a simulation-driven design by providing users with the means of experimenting with different configurations of body sensor networks. However, BodySim does not offer an automatic search for an optimal design; rather, the use and the analysis of the simulated data are entirely left to the user of the platform. Moreover, the project was never released to the public and appears to have been discontinued.

Xia and Sugiura^2^ developed a virtual environment in Unity3D in which a realistic human model can be animated to mimic real-life activities. The platform supports the placement of virtual accelerometers on the human model, which gives the designer the ability to create synthetic datasets. However, the platform does not provide the designer with any analysis tools to assist with the search for an optimal system design (i.e., the number of sensors and their locations to maximize the recognition performance of HAR algorithms). Xia and Sugiura extended their previous work with the inclusion of an optimization tool that is oriented towards searching for a combination of sensor locations to maximize the activity recognition performance^3^. The tool is based on the Cuckoo Search algorithm and serves as a lightweight alternative to dynamic programming. The researchers pointed out that the solutions found by the optimization tool performed equally well in comparison to those that are provided by dynamic programming. The drawback in the previously mentioned studies is their use of complex and expensive motion capture systems to record data for the input of the system. Our work, however, does not require highly accurate input data. Due to its robustness, our pipeline is capable of achieving its goal with imprecise pose estimations from off-the-shelf computer vision methods.

Standalone solutions (i.e., solutions that are not included in a simulation-driven platform) for sensor placement optimization have been proposed. Keally et al.^11^, Cao et al.^10^, and Min et al.^12^ employed a classifier for each possible sensor placement. Thus, they transformed the problem of sensor placement optimization into a classifier selection problem. Yang et al.^9^ employed Markov decision processes to optimize the placements of sensors. Leite and Xiao^8^ devised a deep learning-based optimization algorithm that quantifies the level of redundancy and irrelevancy of each sensor placement. Placements with a high level of redundancy and irrelevancy were excluded.

The previously described studies focused on performing sensor placement optimization on sensor readings. However, our method proposes analyzing the statistics of joint angles. It quantifies the independence and correlation relationships between the joints and uses heuristics to filter out irrelevant and redundant placements. This results in a faster, data-efficient, explainable, and intuitive approach.

## Methods

In this section, an overview of the pipeline is provided, followed by a detailed description of each component.

### System overview

**Figure 2:**
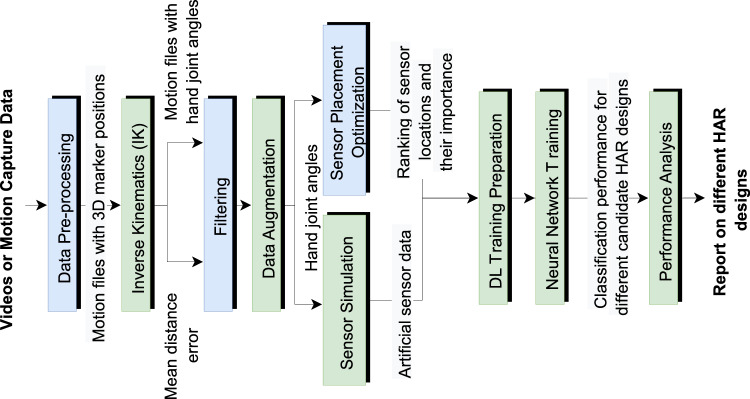
An overview of our system. At the beginning of our pipeline, input data are supplied in the form of videos or motion capture data. When fed with video data, our pipeline utilizes MediaPipe^13^ to estimate 3D positions for hand key-points (i.e., markers). By processing the input data at several stages, our pipeline aims to provide the user with a report of the best HAR designs according to their activity recognition performance and computational costs. The mean distance error that is utilized as input to filter results from the inverse kinematics module is defined in Sect. “Inverse kinematics and filtering”. The modules that are represented in blue represent the novelty of our work. The modules in green are implementations of existing techniques.

Our pipeline, illustrated in Fig. 2, comprises nine distinct steps. Starting with either video or motion-capture-based data as input, the initial step involves data preprocessing, where the data is prepared for inverse kinematics on a human model (the *inverse kinematics* block). The output of this block consists of motion files defining the movement of each joint in the human model. The inverse kinematics also help identify unnatural hand poses violating finger joint constraints, allowing us to filter out poor pose estimations.

The *data augmentation* block enriches the dataset, compensating for any reduction due to filtering, by generating additional motion files from existing ones. All these motion files are used to simulate IMU readings placed at various locations on the human model in the *sensor simulation* block. Simultaneously, an analysis of independence and correlation is conducted in the *sensor placement optimization* block to identify candidate sensor locations and their importance for the classification task.

The artificially generated data are then normalized and segmented into sliding windows in the *DL training preparation* block before being used in neural network training (the *neural network training* block). Multiple proposed HAR designs are trained concurrently at this stage.

Finally, the *performance analysis* block evaluates the activity recognition performance and computational cost of each HAR design, providing the user with a comprehensive report. Computational cost is calculated on a Raspberry Pi microcontroller by determining the memory footprint and inference time of the corresponding neural network for each HAR design. With this pipeline, users need only provide input data and choose from the candidate optimal HAR designs based on the generated report.

### Motion data capture and preprocessing

**Figure 3:**
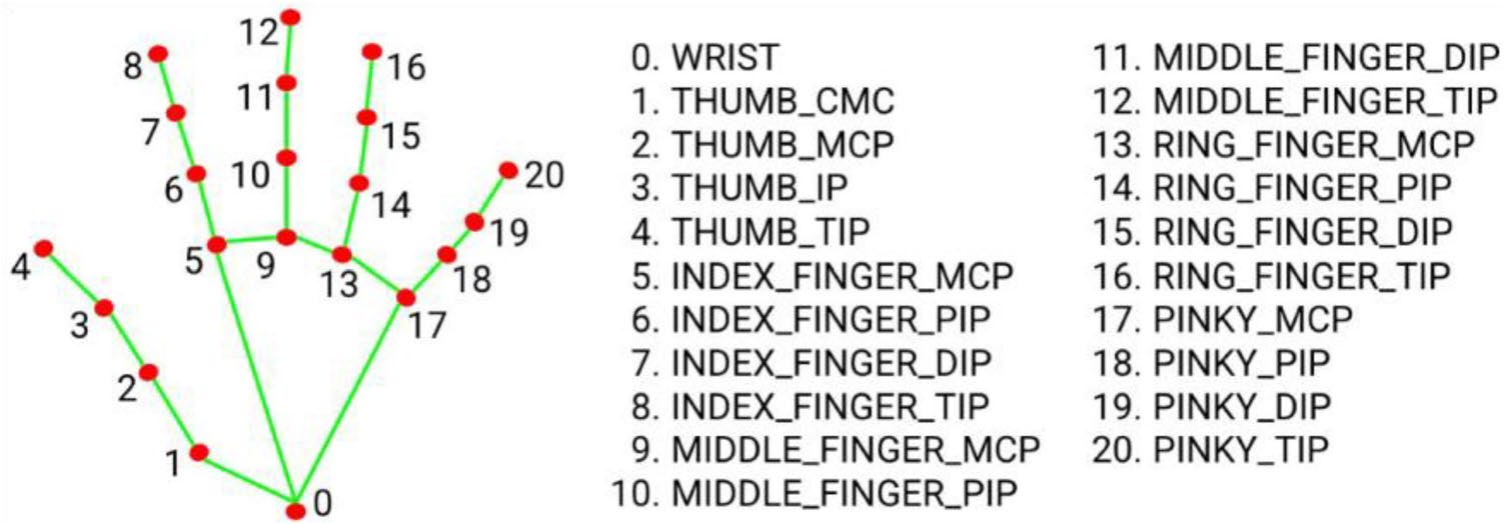
Twenty-one key-points of the InterHands, CMU Panoptic, and Rendered Handpose datasets. These also correspond to the key-points that were extracted by the MediaPipe hand module^34^.

The first step of the simulation system from Fig. 2 requires a rich source of human motion data, specifically hand dynamics information. Our platform supports two types of input: motion capture data and video data. The first type of data consists of temporal finger key-points coordinates from a predefined range of activities and gestures. Datasets such as *InterHands, CMU Panoptic, Rendered Handpose* use a common set of 21 key-points that cover the major hand joints (Fig. 3). The key-points are associated with hand joints and fingertips (four points for every finger and one for the wrist or the center of the palm). The underlying technology that is used to estimate the hand shape and position affects the precision of the provided coordinates. The most accurate systems are optical motion capture systems and manual labeling; 3D bootstrapping and IMU sensor-based motion capture involve a certain level of noise.

The second type of input involves extracting 3D pose information from video databases using off-the-shelf deep learning solutions like MediaPipe. Our focus lies on video datasets centered on gestures and manual activities, which can be processed using the MediaPipe hand module to extract key-point coordinates. MediaPipe extracts a total of 21 key-points (see Fig. 3). The precision and usability of extracted coordinates depend on multiple factors, including the video resolution, framerate, hand visibility, and the MediaPipe model’s noise. Videos with low-resolution frames lack details for capturing fine hand movement details, and low framerates disturb the continuity of hand dynamics. Overall, video databases are a rich source of motion data but may yield more noisy hand dynamics in comparison to specialized databases with annotated hand shapes.

For our experiments, we utilized both strategies (i.e., motion capture data and videos) to gather data for the inverse kinematics module. The strategies were tested individually rather than combined, demonstrating the success of our pipeline when using both types of data as input. Hand coordinates from the first strategy were directly input into the pre-processing stage. Video files from the second strategy were first processed using the MediaPipe module, and the resulting coordinates underwent pre-processing. This pre-processing involved three steps: scaling, translation, and rotation, aimed at reducing errors during the inverse kinematics procedure. These errors could otherwise lead to the virtual hand model being unable to accurately reproduce the gestures present in the motion data.

The first step—scaling – ensures that the hand shape contained in the motion data is approximately equal to that of the virtual hand model for each time step. We scaled each hand segment in the motion data (each green segment between two red circles in Fig. 3) such that its length equals that of the hand’s model. The choice to scale the motion data instead of the hand model is related to the fact that the motion data often do not have real human dimensions. This is especially true in cases where the data are obtained from monocular 3D pose estimation algorithms such as OpenPose^19^ or MediaPipe^13^. In such cases, the bone lengths are never constant but vary from time step to time step. Since we always utilized a right-hand model, a mirroring process was applied to the motion data before scaling in case it contained a left hand.

The coordinate system of the extracted 3D hand poses does not correspond to that of the virtual environment and, very importantly, may be arbitrary and vary from video to video and even from time step to time step. The reason for such arbitrariness is the inherent nature of estimating 3D information from monocular videos. To solve this, we apply translation and rotation transformations to the data before its utilization in the inverse kinematics module. Without such transformations, the inverse kinematics module produces virtual motions largely distinct from the real ones captured on video.

In the translation step, the 3D coordinates in the motion data were moved so that the point of interest located on the wrist (Location 0 in Fig. 3) coincided with the one on the hand model. The final step—rotation—made use of the Kabsch algorithm^35^ to find an optimal rotation matrix which, when applied to the motion data, aligned the hand that was made using the motion data with the hand model. The Kabsch algorithm expects two paired sets of points as input. We utilized the points of interest in the motion data and their corresponding counterparts in the hand model as the required input.

For the pre-processing, both strategies for hand motion data capture were used to provide coordinates for the inverse kinematics. That is, the output of the preprocessing step comprised refined motion files that contained 3D marker positions. We report the effects of different types of input on the quality of the inverse kinematics in 4.2. Note that the user can always quickly visualize the result of the pre-processed input data in OpenSim to ensure that the sensor placement optimization algorithm is receiving reasonable data for making informed decisions on the optimal design, and thus be confident about the final design despite the use of noisy input data.

### Inverse kinematics and filtering

**Figure 4:**
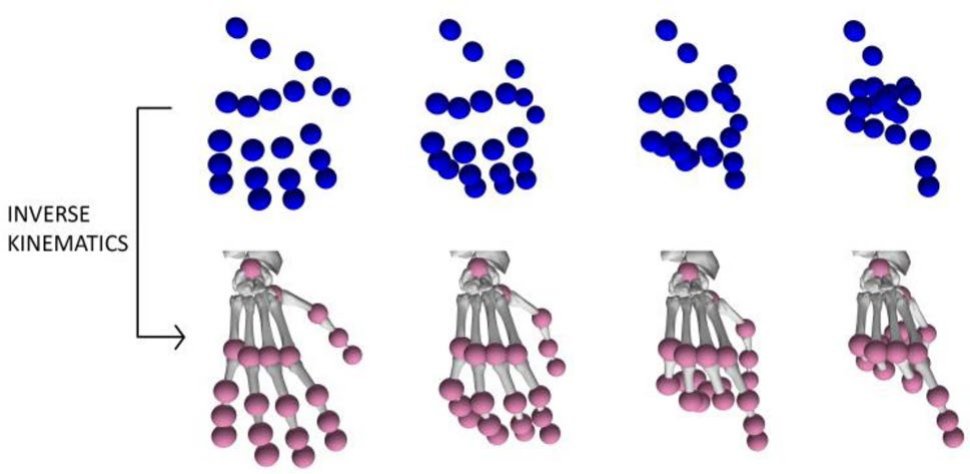
First row: human motion data containing 3D coordinates of various locations of visual markers on the hand (depicted in blue). Second row: the result of the inverse kinematics module where the values for the hand joints are placed so that the hand reproduces the gesture seen in the motion data.

The motion files described previously were used as input for the inverse kinematics module (Fig. 4). The goal of this module was to transform the 3D coordinates in the motion files into hand joint angles (an example is presented in Table 1). After the data were pre-processed, the inverse kinematics algorithm was executed. The optimization problem, for each time instant, finds an optimal set of values for the hand joint angles so that it minimizes the mean square error between the points of interest on the hand model and their corresponding counterparts in the motion data. The result for the inverse kinematics module also consisted of motion files. However, instead of containing 3D coordinates for the points of interest in the hand, these motion files contained a time series that represented the angles of each joint modeled in the hand model for each time instant. We represented such time series as *x*_*a,s,f,j*_, where *a* denotes the activity being performed in *x*, *s* the recording session (assuming that the same activity is recorded multiple times), *f* represents a particular finger, and *j* represents a particular finger joint.

**Table 1:** An example of a motion file that was produced using the inverse kinematics module.

Time	INDEX_MCP_FLEXION	INDEX_ABDUCTION	INDEX_PIP	INDEX_DIP
0	12.5531	− 5.4419	8.0263	− 1.4147
0.0333	14.0483	− 6.5076	8.3055	− 1.7582
0.0667	15.2968	− 7.3226	8.5983	− 1.7382
0.1	16.3127	− 7.9190	8.8943	− 0.2064
0.1333	17.1101	− 8.3292	9.1832	2.4983
0.1667	17.7032	− 8.5854	9.4549	6.0583

It was saved as a comma-separated value file. Each column represents a different finger joint, and the first column represents time. The rows contain the angle values for each finger joint. The format was the same for the motion files that were given as output from the data pre-processing module, with the exception that the columns represent the X, Y, and Z axis components of 3D markers.

In Table 1, one example of *x*_*a,s,f,j*_ is all the values of the *INDEX_MCP_FLEXION* column, which represents the angle of the metacarpophalangeal joint of the index finger. OpenSim’s API^6^ was employed to perform the inverse kinematics. OpenSim is an open-source software that is oriented towards the development, simulation, and biomechanical analysis of musculoskeletal structure models. As for the musculoskeletal model, we adopted the upper-extremity model developed by Lee et al.^36^, which incorporates a model of a right hand with 26 degrees of freedom. Each finger is provided with 4 degrees of freedom and the hand is able to rotate and translate in three dimensions.

As discussed in Sect. “Experiment 1: inverse kinematics”, the inverse kinematics module also outputs a quantitative analysis that evaluates the quality of the produced joint angle motion files. Such an analysis contains the mean distance error between the points of interest of the hand model and their corresponding counterparts in the motion data. Based on the premise that a higher distance error is caused by noisy input data, motion files for which such distance errors exceeded a pre-defined threshold were filtered out.

### Data augmentation

Existing datasets contain only a limited number of user examples. Moreover, the filtering procedure that was introduced in the previous step may result in even smaller dataset sizes. To remedy this issue, two different types of data augmentation were applied to the collected motion data. Our goal was to take the provided motion examples and generate variants that were significantly different from the original, but not so much as to change the class of the gesture.

The first type of data augmentation is hand size augmentation. As inverse kinematics yield a time series of joint angles, small changes to the shape of the hand will not change the output class. Our augmentation is based on statistical measurements of the human hand^37,38^. We modeled the distribution of the hand size as a normal curve centered at 19.3 cm with a standard deviation of 1.25 cm. The hand size was measured from the wrist joint to the tip of the middle finger. Based on the study of Manning et al.^39^, the ratio between the index and the ring fingers was modeled as a normal distribution centered at 1.0 with a standard deviation of 0.05. The ratios between the remaining fingers are the same as in the work of Lee et al.^36^.

Gesture augmentation forms the second type of data augmentation. Similar to the hand size augmentation, the gesture augmentation was completed after computing the inverse kinematics stage in Sect. “Experiment 1: inverse kinematics”. This stage was the most convenient for describing the motion as it expresses the data in joint angles instead of spatial points, which limits the degrees of freedom. A simple rotation of the wrist would yield completely different data for fingers in the spatial domain, but with joint angles, the change is minimal. Simple augmentation methods for gestures, such as additive noise in angular displacement, velocity, or acceleration, were not sufficient. Noise in displacement yields unrealistic tremor-like motions, which would simply reflect measurement noise instead of a realistic variation of the same gesture. Added noise in velocity or acceleration, however, can yield a completely different gesture class due to introduced drift when integrating the motion back to poses. Hence, we chose to use an approach that was inspired by Park et al.^40^ and Ma et al.^22^: a time-warping based augmentation. This process comprises two steps.

First, key poses were extracted for the gesture. In this context, a gesture is defined as an ordered set of poses that are covered in a single motion. These poses need not be consecutive ones, and the time taken from one key pose to another can vary. For instance, a thumbs-up gesture would include a neutral pose, which would be followed by a thumbs-up pose and end in a neutral pose again.

Automatic key pose extraction was then conducted using the process that was described by Ma et al.^22^. Their method uses similar assumptions about key-poses and finds the poses as times when the variance of the overall motion changes direction. This happens by computing the traces of the eigenvectors’ covariance matrices, *D* = $\surd Tr(Q{Q}^{T})$, where *Q* is obtained via eigenvalue decomposition from original time series *A*: *A* = *Q*Λ*Q*^−1^. Since *D* is an expression of the variance in the full motion, locating the local minima and maxima can be considered to yield the poses in which the motion as a whole changes significantly^22^. For the minima and maxima extraction, we used a prominence-based peak detection in Python SciPy library^41^ after normalizing *D*. Overall, this was a modification of the original method by Ma et al.^22^ since we focused on the hand instead of the full body and used a different approach for minima and maxima extraction.

Next, we simply multiplied the time differences of key poses with small, bounded random noise and resampled the motion between the poses via the Fourier method^41^. As this method yielded some end effects, we smoothed them using a Butterworth low-pass filter. Finally, the pose-to-pose segments were joined so that the start and end points aligned, which removed large instantaneous jumps from the resulting motion.

This method can yield new motions by adjusting the key poses to a different degree. The resulting motions can be faster, slower, and have slightly different angles at the key poses. Maintaining low enough bounds, [− 0.4, 0.4] in our case, in the random multiplicative time adjustment ensured that the new motion was close to the provided motion and that the gesture class was more likely to be preserved.

### IMU sensor simulation

**Figure 5:**
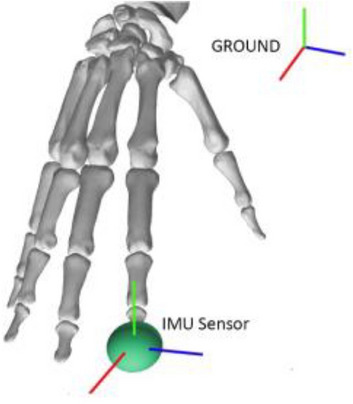
In OpenSim, we modeled an IMU as a marker (represented by the green sphere) that possessed its own reference frame (axes represented by colored lines). The position of the marker and the orientation of the marker’s reference frame were expressed as functions of time in the ground’s reference frame.

Utilizing OpenSim’s API, we modeled each IMU as a marker that was attached to a specific location of the hand model (Fig. 5). The marker was described using two quantities: (1) position and (2) orientation. Since the marker was subject to movements that were imposed by the hand model, these two quantities were a function of time. The position of the marker is represented by a three-dimensional vector *p*, which is expressed in the ground’s reference frame, whereas the orientation of the marker is characterized by a unit quaternion *q* that defines a rotation to the ground’s reference frame in such a way that it aligns with the marker’s reference frame.

For every motion file that was generated by the inverse kinematics module, we obtained the IMU’s quantities *p* and *q* for each time instant. The acceleration of the IMU was numerically computed by taking the second derivative of *p* with respect to time (Eq. (1)). The angular velocity of the IMU in the IMU’s reference frame *ω* is defined as in Eq. (2)^29^.

1 $${\varvec{a}}=\frac{{d}^{2}{\varvec{p}}}{d{t}^{2}}$$

2 $${\varvec{\upomega}}=2 \, {{\varvec{q}}}^{\boldsymbol{*}}\times \frac{d{\varvec{q}}}{dt}$$

where *q*^∗^ is the conjugate of *q*, the symbol × expresses the quaternion product, and $\frac{d{\varvec{q}}}{dt}$ is calculated numerically.

Alternatively, if *R* is the rotation matrix that transforms quantities expressed in the ground’s reference frame to the IMU’s reference frame, the angular velocity of the IMU in the ground’s reference frame can be obtained from the skew-symmetric matrix defined in Eq. (3).

3 $$S\left({\varvec{\upomega}}\right)=\frac{dR}{dt}\cdot {R}^{T}= \left[\begin{array}{ccc}0& {-\upomega }_{\text{z}}& {-\upomega }_{\text{y}}\\ {\upomega }_{\text{z}}& 0& {-\upomega }_{x}\\ -{\upomega }_{y}& {\upomega }_{\text{x}}& 0\end{array}\right]$$

where $\frac{dR}{dt}$ is the derivative calculated numerically with respect to time of the rotation matrix *R*, *R*^*T*^ is the transpose of *R*, and *ω*_*x*_*, ω*_*y*_*, ω*_*z*_ are the components of the angular velocity in the *x, y, z* axis, respectively.

The IMU sensor readings were simulated utilizing MATLAB’s *imuSensor* function, which requires three quantities expressed in the ground’s reference frame as input: (1) the acceleration of the IMU ***a***, (2) the angular velocity of the IMU *q* × *ω* × q^*^, and (3) the orientation of the IMU ***q***. It is also possible to define a set of error-related properties of the accelerometer, gyroscope, and magnetometer such as the measurement range, resolution, constant bias, axes misalignment, noise density, bias instability, random walk, temperature bias, and temperature scale factor. A description of these properties and how they mathematically influence the error-free readings of the IMU is provided in MATLAB’s documentation^32^. In short, given the error-free quantities (e.g. acceleration), MATLAB applies a few filters with different transfer functions to simulate random error signals, as well as multiplicative and additive signals to simulate systematic errors. In our work, we used all the error quantities that are provided by MATLAB to deliver a more realistic simulation.

### Sensor placement optimization

Narrowing down the sensors is particularly meaningful in cases of on-device processing and analysis in which the microcontroller responsible for processing the data cannot handle tons of input. For instance, an Arduino Uno board supports less than 6 IMU sensors that are connected to the I2C bus before exceeding the physical characteristics of the bus. Utilizing a higher number of sensors in an activity recognition algorithm also signifies higher memory, processing, and power consumption. Reducing the number of sensors is an effective strategy for lowering the processing costs and power consumption in a predictable, linear manner^8^. Furthermore, a smart glove with more sensors can be cumbersome to wear and can limit the movements of the users. Finally, the higher the number of sensors, the greater the probability of at least one sensor malfunctioning. A malfunction in a sensor can significantly shift the data distribution and cause the recognition algorithm to perform poorly.

Here, we utilized the Pearson coefficient to quantify the dependence between the joints of the same finger and between the joints of distinct fingers. Our goal was to select sensor locations based on the analysis of hand joint independence and correlation. As will be seen, our purpose was to obtain indications of a sensible set of sensor locations that minimized redundant and irrelevant information for activity recognition. The Pearson correlation coefficient, which is defined in the interval [-1, 1], measures the degree of a linear relationship between two distinct signals. It is defined as the quotient between covariance of the signals and the product of their standard deviations (Eq. (4)).

4 $$r\left(x,y\right)=\frac{{\sum }_{k}\left({x}_{k}-\overline{x }\right)\left({y}_{k}-\overline{y }\right)}{\sqrt{{\sum }_{k}{\left({x}_{k}-\overline{x }\right)}^{2}}\sqrt{{\sum }_{k}{\left({y}_{k}-\overline{y }\right)}^{2}}}$$

where *x* and *y* are discrete signals whose quantities are indexed by *k*. *x̅* and *y̅* represent the average of *x* and *y*. A value of *r*(*x,y*) above 0 indicates that *x* and *y* present a positive correlation; that is, as *x* increases, so does *y*. A negative correlation exists between *x* and *y* for *r*(*x,y*) < 0. In the case of *r*(*x,y*) = 0, *x* and *y* are not linearly correlated.

**Figure 6:**
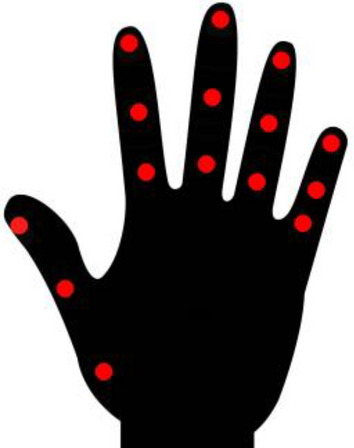
Pre-defined sensor locations (in red; i.e., prior to the sensor selection).

Researchers have proven that sensors that are closely located to each other and lack relative motion convey the same information^8^, which results in redundancies. In our case, this signified that a sole sensor is sufficient for each finger bone. Based on this insight, we started with a pre-defined set of sensor locations, as illustrated in Fig. 6. For each pre-defined sensor location, we aimed to determine a score in the range of 0 to 1 that assessed its importance for the activity recognition task. A score of 0 indicated that the placement of an IMU at that specific location was completely dispensable, and vice-versa. Ranking the sensor locations according to this score provided us with the means to perform sensor selection.

**Figure 7:**
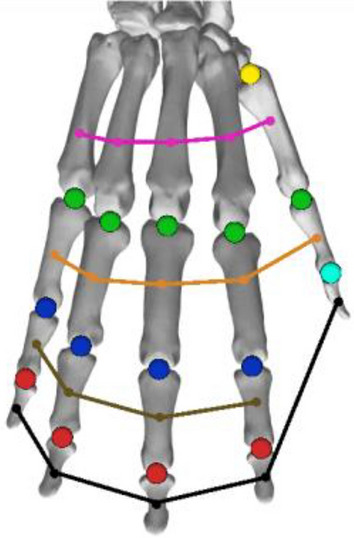
Finger bones and joints.

Sensors that are subject to a wider amplitude of motion are more likely to convey more relevant information for activity classification^8^. In our case, these sensors were located on the extremities of the hands (i.e., the fingertips). Therefore, for a finger *f*, we start by assigning the importance *s*_*f,DIP*_ of a sensor placed on the distal phalanx of *f* as 1. For the middle phalanx of *f*, the score *s*_*f,MIP*_ is given as the *inter-joint independence* between the distal interphalangeal (DIP) and proximal interphalangeal joints (PIP), that is *s*_*f,DIP*_ = *G*_*f,DIP,PIP*_ in Eq. (5). Finally, the score for a sensor located on the proximal phalanx was determined as the product of the PIP-MCP (metacarpophalangeal) inter-joint independence between the PIP joint and the DIP-MCP inter-joint independence (i.e., *s*_*f,PIP*_ = *G*_*f,PIP,MCP*_ ·*G*_*f,DIP,MCP*_). The thumb was an exception since it does not have a middle phalanx, so a related score was not available. Instead, the score that was relevant to the proximal phalanx was given as the inter-joint independence between the interphalangeal (IP) and MCP joints. The product of the IP-CMC (carpometacarpal) and MCP-CMC inter-joint dependencies formed the score for the metacarpal bone of the thumb. Figure 7 illustrates the finger bones and joints.

5 $${G}_{f,{j}_{1},{j}_{2}}=1-\Vert \frac{1}{A}\sum_{a}^{A}\frac{1}{{S}_{a}}{\sum }_{s}^{{S}_{a}}r\left({x}_{a,s,f,{j}_{1}},{x}_{a,s,f,{j}_{2}}\right)\Vert$$

where *A* and *S*_*a*_ denote the number of activities and recording sessions for activity *a*, respectively. The average of *r*(*x*_*a,s,f,j*1_*,x*_*a,s,f,j*2_) (defined in Eq. (4)) across recording sessions and activities ranges from − 1 to 1. However, we were only interested in its absolute value. *G*_*f,j*1*,j*2_ ranges from 0 to 1 and designates a score for a sensor placed on the bone between joints *j*_1_ and *j*_2_. Note that each activity was weighted equally in the averaging of Eq. (5). This ensured that a possible activity imbalance that might have been present in the dataset did not result in biased scoring. A small value of the inter-joint independence denoted that the motion of one joint was well predicted from the motion of another joint. In such a case, the original two degrees of freedom were reduced to a sole degree of freedom, which rendered one sensor redundant.

The scores as they were previously defined did not contain any information on the relative importance of one finger with respect to another. To include such information, we propose multiplying the score *s*_*f,j*_ with an additional value termed as *inter-finger independence* (Eq. (7)). Before defining this quantity, we introduce a measure to quantify the redundancy of a certain finger in comparison to others.

A finger was deemed redundant if its motion could be well predicted from the motion of another finger. We measured the degree of redundancy of a finger *f* in an activity (or gesture) *a* by first finding the average Pearson correlation coefficient between *x*_*a,s,f,j*_ and each *x*_*a,s,f*_′_*,j*_, with *f*^‘^ ≠ *f*. The average was computed over all joints *j* and recording sessions *s*. Each of these computed values represented how much the motion of the finger *f* was redundant to the motion of *f*^′^ for the activity *a*. Taking the maximum of these values denoted how *f* was redundant in *a*. The described *finger degree of redundancy* is mathematically defined in Eq. (6).

6 $${D}_{f,a}=\underset{{f}^{\prime}\in {\mathbb{F}}_{f}}{\text{max}}\Vert \frac{1}{J}{\sum }_{j}^{J}\frac{1}{{S}_{a}}{\sum }_{s}^{S}r\left({x}_{a,s,f,j},{x}_{a,s,{f}^{\prime},j}\right)\Vert ,$$

where *J* is the number of joints, *f* is the set of all fingers with the exception of *f*.

Again, the absolute value of the average of *r*(*x*_*a,s,f,j*_*,x*_*a,s,f*_′_*,j*_) is taken since we are not interested in whether or not the correlation it defines is positive or negative.

The minimum value of *D*_*f,a*_ across activities—i.e. min_*a*∈_ *D*_*f,a*_—denotes the degree of redundancy of a finger *f* in the best case scenario. By subtracting this quantity from 1, we define the *inter-finger independence* (Eq. (7)) of a finger *f*.

7 $$H_{f} = { 1} - \;\mathop {\min }\limits_{{a \in {\mathbb{A}}}} D_{f,a} .$$

The overall importance score for a sensor placed on a bone between joints *j*_1_ and *j*_2_ was given as in Eq. (8). If the bone was the distal phalanx, its score was calculated as in Eq. (9). Higher scores represent higher importance. Therefore, the importance scores can be used to find a set of *n* sensors that will most likely lead to better classification performance.

8 $$I_{{f,j_{{1}} ,j_{{2}} }} \, = G_{{f,j_{{1}} ,j_{{2}} }} \cdots H_{f} ,$$

9 $$I_{f,DIP} = H_{f} .$$

Utilizing the ranking from the greatest to the lowest importance score, we generated *k* different sets of sensors where the *i*-th set is composed of the sensors with the *i*-highest importance scores. Each of these sets of sensors can be quantitatively evaluated by training an activity classifier with simulated IMU data generated from them and measuring the classification performance of the classifier. The computational cost associated with utilizing a particular set of sensors can also be calculated by quantifying the memory footprint and inference time of the neural network that was trained for the set. Therefore, at the end of our pipeline, the user is provided with a report that contains the classification performance and the resource utilization of a few sets of sensors. The user is expected to utilize such a report to choose a set of sensors that meets their requirements regarding the trade-off between accuracy and cost. Note that, as mentioned in Sect. “Introduction”, the use of finger joint angles leads to a more tractable and simpler approach to selecting the placement of sensors.

### Training and evaluating classification models

The ranking of sensor locations and their importance, as provided by the sensor placement optimization module, was used to generate candidate optimal HAR designs. An individual network for each of these designs was trained with the artificially generated data. Before the training, the data were prepared by applying normalization and segmenting them into sliding windows. After the training, a report on each HAR design was provided. Such a report contained the recognition performance of each neural network, as well as its memory footprint and inference time on a Raspberry Pi microcontroller.

### Code implementation

Our pipeline was implemented in Python 3.8.8 by utilizing OpenSim’s API 4.3 for the operations involved in the modules of inverse kinematics and sensor simulation, in which MATLAB 2021a was also employed. TensorFlow 2.7.0 was employed to perform neural network training and inference. The code was executed on an Intel 11,700 CPU and an NVIDIA RTX 3070 GPU with 8 GB of RAM. As for the neural network for gesture recognition, we opted for InnoHAR’s architecture^42^. The choice of hyperparameters was identical to that of the original paper, except for the number of filters. This hyper-parameter was chosen to be 128 for the experiments in Sect. “Data augmentation” and 64 + 8 · num sensors for those in Sects. “Data augmentation" and “Case study: smart glove design”.

Before the neural network training, the data were normalized to zero mean and unit variance. We also followed the conventional sliding window approach for segmentation^43,44^; each sliding window was annotated with the activity (hand gestures, in our case) that was present throughout most of the window. We chose the length of the sliding windows for all datasets to be 0.5 s with an overlap of 80%. This choice was made considering the short duration of the hand gestures included in the datasets. The training, validation, and test sets for the neural network training had the proportions of 0.6, 0.2, and 0.2, respectively. When training and testing the neural network with augmented data, we took special care to prevent data from leaking from the validation or test sets into the training set. The neural network was trained for 20 epochs. At the end of the training, the model with the best F1-score (to be defined in Sect. “Experiment 2: sensor simulation”) in the validation set was selected to infer the samples of the test set and provide a final test F1-score. The previously mentioned hyper-parameters may be easily modified by the user of the pipeline.

## Experimental results

Our pipeline is composed of diverse blocks that are arranged in a sequential matter. Each block contains intrinsic errors that eventually accumulate until the output of the pipeline. In this section, before analyzing the final results of our pipeline, we study each block individually to understand how they affect the output. Even though the quality of the input data is not tested, we discuss it and its effects on the pipeline. Before the evaluation, we describe the datasets that we utilized in the experiments.

### Datasets

We utilized three datasets for the experiments. Two of the datasets were composed of monocular videos, whereas the other one consisted of data obtained from a motion capture system.

*InterHand2.6 M*^45^ is a large-scale dataset of labeled single and interacting hand frames that are placed under various poses from multiple subjects. It includes 85 classes of movement sequences (2.6 million frames in total): 53 peak poses in which a certain hand shape is reached from a neutral position and 32 ranges of motion sequences in which hands perform conversational gestures. To obtain the key-point coordinates, we used a hybrid strategy that includes manual labeling, triangulation from 2D coordinates to 3D using multiple cameras, and an automatic machine annotator (a trained 2D key-point detection model). This results in a rich source of motion data with various hand poses and gestures, which made the InterHand our most reliable and accurate source of motion data. We used the following types of sequences in our experiments: opening and closing the hand, spreading the fingers apart, performing a thumbs-up gesture, and performing the rock’n’roll salute.

*First-Person-Action*^46^ is a collection of first-person hand actions that incorporate 3D hand poses. It covers 45 daily hand action categories and object manipulations by providing RGB-D video sequences (˜100 K frames) and 21 key-point coordinates that are extracted via a self-developed motion capture system. We used solely the key-points coordinates from this dataset as a source of naturally occurring hand poses and motions. The hand coordinates of the First-Person-Action dataset are less precise in comparison to InterHands due to the limitations of the motion capture system. We used the following types of sequences in our experiments: writing, opening and closing a juice bottle, opening and closing a milk carton, opening and closing a peanut butter jar, and squeezing a sponge.

*IPN Hand*^47^ is a video dataset with more than 4000 gesture instances from 50 subjects. Thirteen gesture types represent dynamic interactions with a touchless screen and last from 0.5 to 1 s. The dataset consists of videos that have been recorded with a front-facing camera with a relatively high resolution (640 × 480, 30fps), which made it a suitable candidate for our motion from the video module. We used the IPN Hand dataset to extract hand motions from the videos by extracting the hand key points with the help of MediaPipe. The following gestures were chosen: clicking with one finger, clicking with two fingers, throwing the hand upwards, throwing the hand down, throwing the hand left, throwing the hand right, opening the fingers twice, double-clicking with one finger, double-clicking with two fingers, zooming in, and zooming out.

The datasets cover different scenarios of extraction of hand motion information. Table 2 presents the sample sizes that were used in our experiments. The datasets with precise dynamic hand coordinates are more sparse, with 20–30 samples per gesture. On the contrary, video datasets provide more hand information with a high level of noise (˜200 samples per gesture).

**Table 2:** Hands dataset statistics.

Dataset	Type of data	# of gestures	Samples per gesture
InterHand^45^	Precise 3D hand joints coordinates	4	26
First-Person-Action^46^	Estimated 3D hand joints coordinates	5	24
IPN Hand^47^	RGB videos	11	200

### Experiment 1: inverse kinematics

The first experiment consisted of evaluating the quality of the results provided by the inverse kinematics module. Since the aim of the inverse kinematics module is to find optimal hand joint angles that minimize the errors between the marker positions in the input data and virtual hand model, we evaluated the results in terms of the marker positioning errors and the constraint violations of hand joints. The former metric was used as the average and standard deviation of the distance error (in millimeters) between the marker positions from the input and output data (i.e., the results of the inverse kinematics module). The average was computed over all the time steps and all the markers. The latter metric was calculated (1) as the probability (or ratio; in percentage) of constraint violations that were seen in the hand joint angles of the output data and (2) as the average and standard deviations of the constraint violation magnitude, which measures in degrees how much a joint has traveled past its normal operation range.

#### Results

Table 3 details the results of the inverse kinematics module for four distinct datasets: InterHand^45^, First-Person-Action^46^, and the filtered and unfiltered versions of IPN Hand^47^. In the filtered version of IPN Hand, we selected the 30 best takes from the unfiltered (original) IPN Hand dataset in terms of average marker distance error for each of the 10 gestures. Before discussing the results for each dataset, we describe the causes that we attributed to the flaws in the inverse kinematics module.

1. Inverse kinematics consist of a constrained optimization problem in which joint angles are found to minimize an objective function that accounts for the average marker distance error while penalizing solutions that violate the constraints. Nonoptimal solutions are common and will always lead to errors. Furthermore, the penalty component of the objective function only attempted to avoid constraint violations and did not necessarily eliminate the possibility of such violations.
2. The input data—especially if obtained from 3D pose estimations from monocular videos—were prone to contain joint constraint violations. The optimization procedure of the inverse kinematics module did not completely avoid such violations from being reproduced in the virtual model. When it did, it did so at the cost of a higher marker distance error.
3. The placement of the markers in the virtual hand model did not perfectly reproduce their placement in the input data. For instance, in the input data, it was often the case that some markers were located on top of muscles. Since it was not possible to obtain exact measurements of the muscles, it was also not possible to accurately reproduce the same marker locations in the virtual model. On top of that, in real life, markers can move due to muscle contraction or relaxation. Such movements were never considered in the virtual model. These subtle differences between the input data and the virtual model led to errors that were reflected in the results of the inverse kinematics.
4. The scaling process (which is described in Sect. “Inverse kinematics and filtering”) cannot guarantee a perfect agreement between the handshape of the input data and that of the virtual model.

**Table 3:** Evaluation of the inverse kinematics results in terms of the metrics defined in Sect. “Experiment 1: inverse kinematics”.

Dataset	Avg. MDE	Std. MDE	CVR	Avg. CVM	Std. CVM
InterHand^45^	5.54 mm	4.04 mm	15.3%	13.26º	11.61º
First-Person-Action^46^	8.57 mm	7.04 mm	8.2%	27.48º	36.24º
Filtered IPN Hand^47^	6.37 mm	4.56 mm	16.1%	28.32º	24.59º
Unfiltered IPN Hand^47^	11.91 mm	9.54 mm	22.3%	55.35º	45.14º

*MDE* marker distance error, *CVR* constraint violation ratio, *CVM* constraint violation magnitude, *Avg.* average, *Std.* standard deviation.

##### InterHand

**Figure 8:**
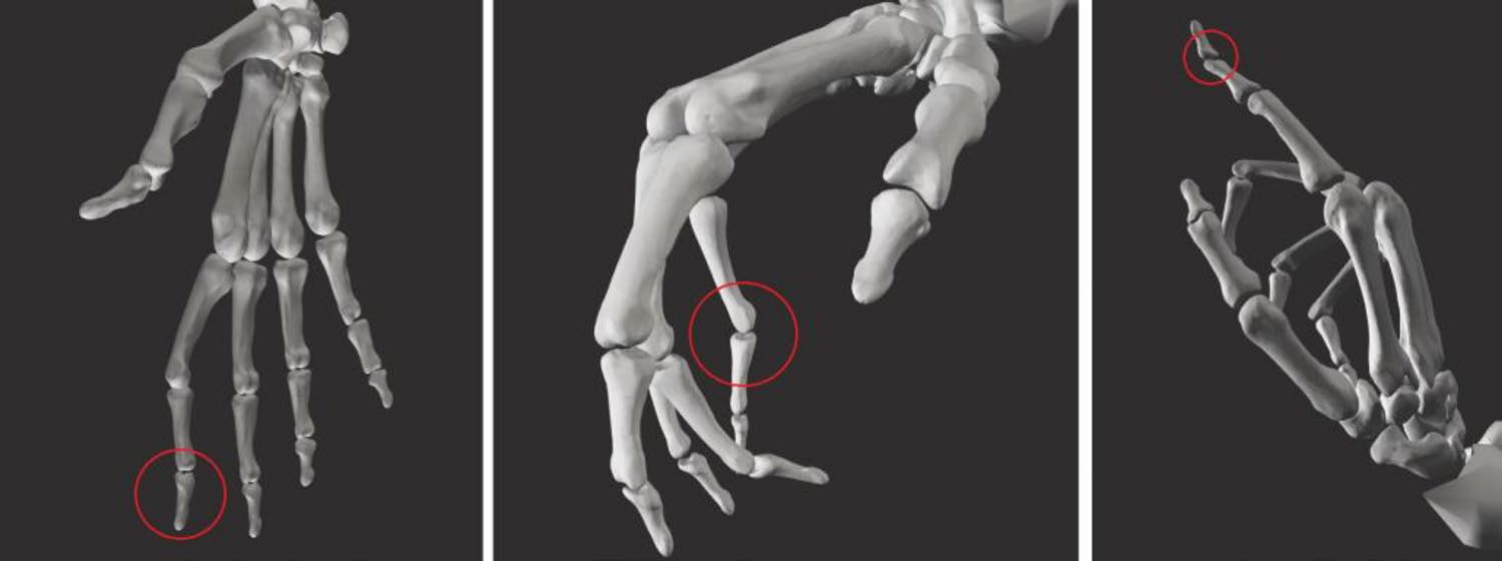
Some examples of constraint violations. Left: A constraint violation of approximately 9º in the index DIP joint InterHand dataset. Center: The constraint of the PIP joint in the pinky finger is violated by roughly 23° (First-PersonAction dataset). Right: The index DIP joint bends backward, exceeding its limit by an angle of 27.5° (filtered IPN Hand dataset).

For the InterHand dataset, a rather small average marker distance error below 5 mm was obtained, which contributed to realistic motions of the hand model. With respect to the constraint violations, although they were observed in approximately one-eighth of the data, the magnitude of the violations was relatively small and hardly noticeable in image visualizations (Fig. 8). A simple procedure of clipping joint angles outside the limits of the constraints resolved the issue while retaining the original nature of the finger motions.

##### First-person-action

The authors of the First-Person-Action dataset^46^ reported an estimated hand pose error in the annotations of 10–20 mm due to imprecise hand pose estimations. This reduced quality of human motion data signifies a higher incidence of unnatural hand poses, which in turn results in a larger MDE or CVR. Hence, the inaccuracies in the input data—i.e., hand pose estimations—were the main cause of the larger errors in the inverse kinematics. A lower incidence of constraint violations was associated with the fact that the majority of the hand gestures — except for the activity of squeezing a sponge—in this dataset consisted of finger joint angles that resided further from the limits.

##### IPN hand

The unfiltered IPN Hand dataset exhibited the worst results in terms of all the considered metrics since the estimated 3D hand poses were obtained (through the MediaPipe tool^13^) from monocular videos. It is often the case that the handshape estimations output by MediaPipe vary at each video frame and that bones experience significant differences in length. Nevertheless, the filtered version of this dataset presented surprisingly decent results with an average MDE close to more sophisticated datasets in terms of annotation quality (such as InterHand). Without the data pre-processing transformations, the errors in the inverse kinematics module were extremely large (i.e., in some cases with physically impossible hand poses).

In conclusion, the results of the inverse kinematics can be interpreted as a strong indication of the quality of the 3D pose estimation, which is affected by the quality of the videos and the precision of the hand recognition module. When the input data significantly violate realistic joint constraints of the virtual hand, we observe larger errors in terms of the MDE metric and the number of constraint violations. We proposed to filter the video data based on these two metrics and use it as a data cleaning policy that improves the quality of the inverse kinematics and sequentially the sensor simulation.

### Experiment 2: sensor simulation

**Figure 9:**
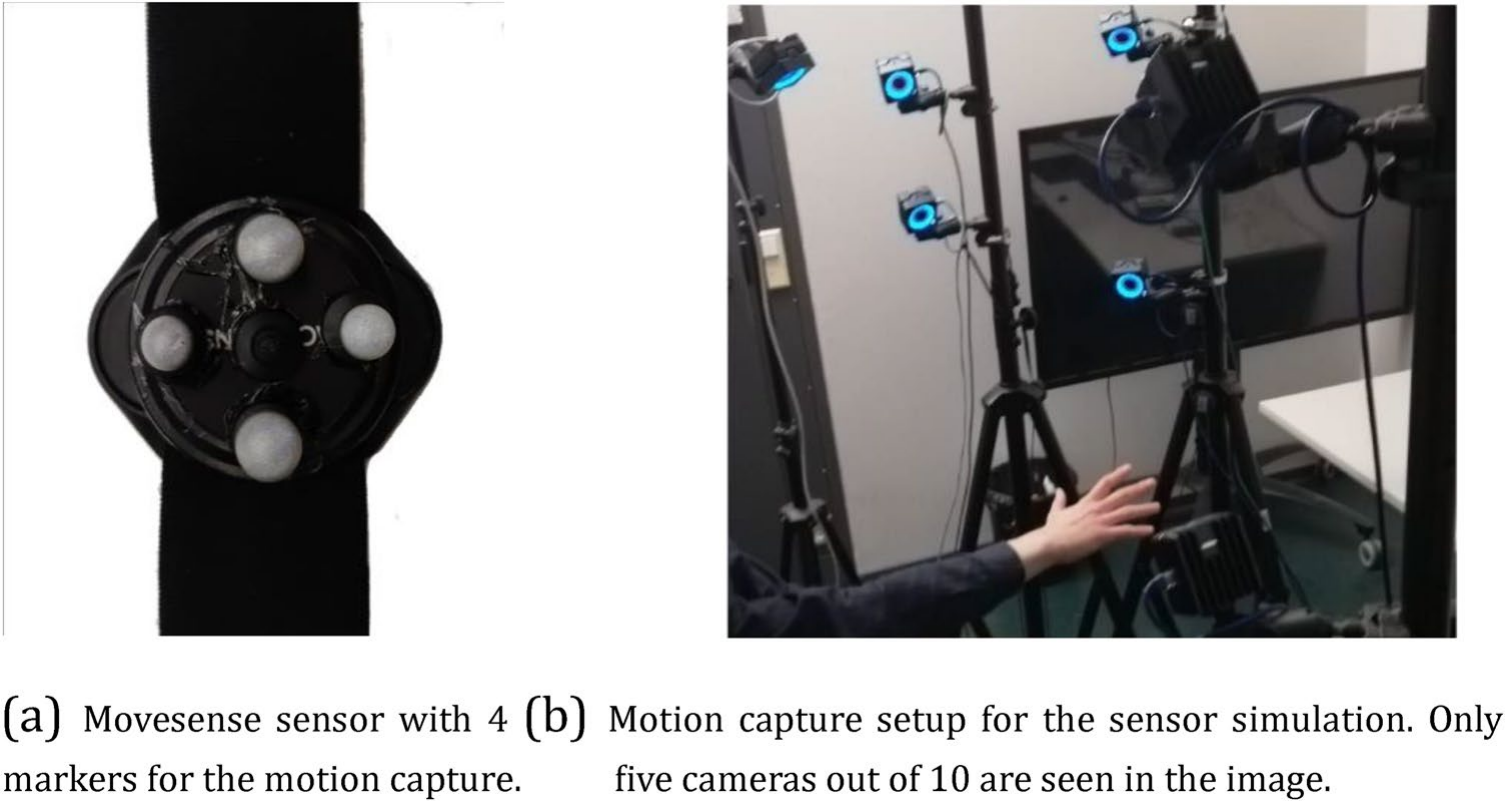
The setup that was utilized to collect data for the evaluation of the sensor simulation block.

To evaluate the sensor simulation block that is introduced in Sect. “IMU sensor simulation”, we performed an experiment using a Motion Capture (MOCAP) system with 10 OptiTrack cameras and a Movesense sensor (Fig. 9a and b). The Movesense device was employed to collect ground truth accelerometer and gyroscope data from a series of movements. The recording session lasted 120 s (30 s. for random translational motions, 30 s. for random rotational motions, 30 s. for chaotic movements, and 30 s. for no movement). At the same time, the MOCAP system recorded the trajectory followed by each of the four markers that were placed on the Movesense device. Due to the occasional losses of the tracking of the markers, 20 s of the trajectory data had to be discarded. The trajectory data were converted into an estimated position and orientation of the IMU for each time instant. The position of the IMU was approximated to be located at the center of the markers. Our evaluation happened in terms of the magnitude of the accelerometer and gyroscope readings—that is, the square root of the sum of each axis component squared (sqrt(*x*^2^ + *y*^2^ + *z*^2^)). Evaluating these *x*, *y*, and *z* axis components individually would require knowledge about where each axis points to.

Since the magnetometer readings were only dependent on the orientation of these axes and since their magnitude was constant, we deemed the evaluation of this sensor modality unnecessary. The data from the two aforementioned modalities were later synchronized and processed using MATLAB’s sensor simulation tool to produce the results seen in Sect. “Experiment 4: sensor placement optimization”.

#### Results

**Figure 10:**
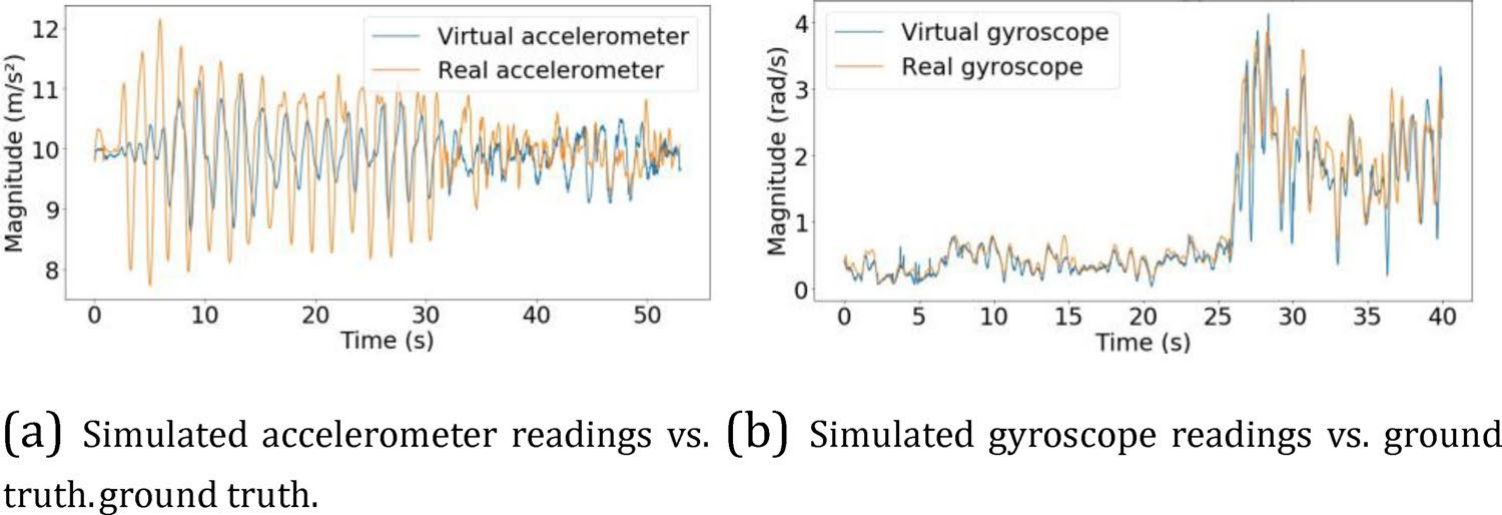
The results of the sensor simulation.

Figure 10a and b illustrate, respectively, the comparison of virtual accelerometer and gyroscope readings with their respective real-world measurements (ground truth). The mean absolute error amounted to 0.641* m/s*^2^ and 0.155* rad/s* for the accelerometer and gyroscope readings, respectively. These errors were mainly attributed to two distinct factors: 1) the mathematical model for these virtual sensors did not thoroughly reflect reality, and 2) the parameters of the mathematical model – such as the scale factor, axis misalignment, and bias – did not accurately represent the real quantities. We made use of the resolution and noise density parameters that were provided by the manufacturer of the device. However, the remaining important parameters (i.e., the random walk, bias, and bias instability) were set to zero since these were not measured by the manufacturers. The larger error observed in the simulation of the accelerometer can be attributed to the first described factor being more pronounced for this modality (possibly due to manufacturing or aging factors).

The quality of the data that was utilized for the generation of virtual sensor readings was a third factor that was associated with the simulation errors. However, its contribution to the imperfect results was minor since the method that was utilized to collect the data (i.e., motion capture) delivers highly accurate measurements. These errors—especially the simulation gyroscope error—were certainly small, and other researchers have previously demonstrated^4,30,31^ that classifiers mostly ignore differences that arise from employing simpler or more thorough sensor simulations. Therefore, we deemed that errors in the sensor simulation led to negligible or null errors at the end of the pipeline. Although our evaluation of the sensor simulation is limited with respect to the set of motions utilized, it serves as a proof-of-concept step. Indeed, the observed results indicate the applicability of the proposed method for simulating sensor readings in hand manipulation scenarios.

### Experiment 3: data augmentation

We evaluated the effectiveness of the dataset augmentations that were provided by the methods discussed in Sect. “Data augmentation” by observing the classification performance of the neural network as the amount of augmented data in the dataset increased. That is, we performed sensor simulations on the joint angle motion files that were produced by the data augmentation module and trained a neural network on such data. The weighted average F1-score was employed as the metric (Eq. (10)) to evaluate the classification performance. This is a widely used metric in HAR since it accounts for class imbalance, which is commonly seen in datasets for HAR.

10 $${F}_{1}=\frac{2T{P}_{i}}{2T{P}_{i}+F{P}_{i}+F{N}_{i}},$$

where *TP*_*i*_, *FP*_*i*_, and *FN*_*i*_ denote, respectively, the number of true positives, false positives, and false negatives for a certain class. Each class is weighted according to its number of samples to obtain a single measure that encompasses all classes.

#### Results

Table 4 exhibits the evolution of the classification performance when the amount of augmented data increases in the dataset. One can observe that the proposed augmentation could considerably improve the generalization performance of the classifier. These results provide evidence that including distinct physical characteristics and varying paces in the dataset is utterly beneficial. The reason for this is that when the neural network is presented with input data of varying physical and pace characteristics, it learns to ignore such information in the feature extraction layers and thus delivers only information that is necessary for activity recognition to the classification layers.

**Table 4:** The classification performance with varying amounts of artificial data in the dataset.

Dataset	Hand size aug					Gesture style aug			
	0%	5%	10%	25%	50%	5%	10%	25%	50%
InterHand^45^	0.921	0.930	0.926	0.923	0.911	0.947	0.928	0.898	0.904
First-Person-Action^46^	0.800	0.828	0.832	0.819	0.828	0.835	0.865	0.790	0.806
IPN Hand^47^	0.874	0.891	0.898	0.900	0.897	0.889	0.916	0.889	0.865

Here, 0% refers to the original setting without augmentation; K% represents a dataset with the inclusion of augmented data in the proportion of k% the original data. For instance, if an original dataset includes 1000 s of data, then a dataset with a 5% data augmentation setting would include 1000 + 50 s.

One can also observe that the gesture style augmentation outperforms, even if by a small margin, the hand size augmentation. Finally, applying both augmentation methods (Table 5) is more effective than only applying hand size augmentation, but it is less effective than gesture style augmentation. Therefore, we observed that it is sufficient to only perform gesture style augmentation. Since we are dealing with black-box models and input data of massive sizes, finding reasons to explain these observations may not be possible.

**Table 5:** The F1-scores when both types of augmentation are present, each of which is presented according to the proportions of 5%, 10%, and 25%.

Dataset	Both augmentations			
	5%	10%	25%	Best
InterHand^45^	0.908	0.919	0.929	0.908
First-Person-Action^46^	0.874	0.814	0.814	0.814
IPN Hand^47^	0.903	0.893	0.871	0.872

“Best” signifies that data from each augmentation method was included in their best proportions (e.g., for InterHand, 5% and 5% for hand size and gesture style augmentations, respectively).

Notice that the performance degradation observed with higher levels of augmented data is not attributed to unrealistic augmented motions. Hand-size augmentation carefully considers three statistical studies^37–39^ on human hand size to ensure that the generated sensor data remains realistic. Gesture style augmentation introduces variation in the speed of hand motion, which is carefully limited to maintain realistic movements. Additionally, the datasets do not include classes that are distinguishable solely by the speed of motion; instead, the classes are distinguishable by the pattern of finger movements.

The reason for the performance degradation due to high levels of augmentation can be explained as follows. The aim of adding the augmented data is to enrich the training distribution and, as a consequence, improve generalization. Since the variation introduced by the data augmentation techniques is bounded, there will be a point where additional augmented data no longer enriches the training distribution. Instead, it will introduce mostly repetitive samples into the training set, potentially leading to overfitting. For our presented augmentation techniques, we recommend using 5% to 10% augmented data, regardless of the neural network architecture used.

### Experiment 4: sensor placement optimization

The purpose of this experiment was to validate our sensor placement optimization algorithm. To evaluate different sensor placements, we utilized the recognition performance of a classifier—the F1-score as defined in Eq. (10)—that was trained on the artificially generated sensor data from the particular set of sensors. Augmented data were not utilized here since they were not needed, and the selection results did not depend on them. We also reported the values of resource consumption in terms of memory footprint (in MB) and inference time (in ms) on a Raspberry Pi 4 B microcontroller while running inference on the classifier with different numbers of sensors.

#### Results

**Figure 11:**
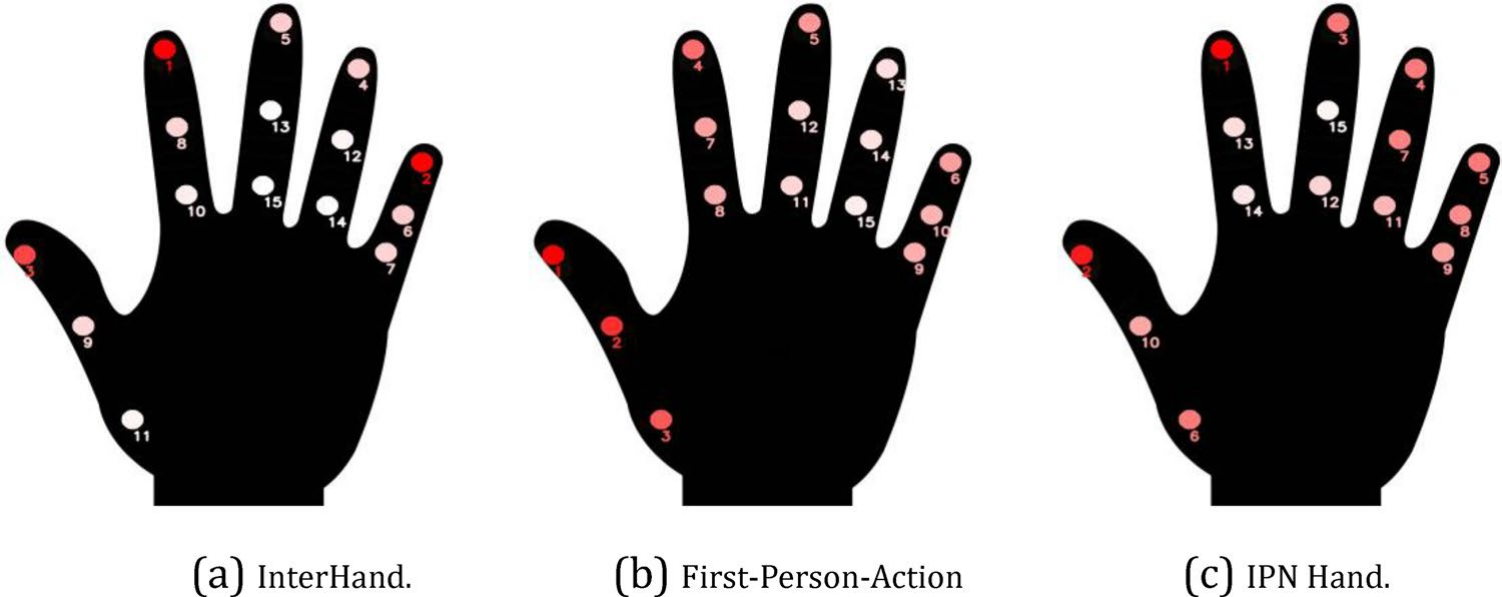
Results of our sensor placement optimization algorithm. Sensors depicted with a more saturated red color denote higher importance. The numbering also indicates the importance, with 1 and 15 expressing the most and the least important sensor, respectively.

Figure 11 depicts the results of our sensor selection algorithm. In the case of the InterHand dataset, we observed that the tips of the thumb, index, and pinky fingers were ranked as the most important locations due to the activities of giving a thumbs up and making the rocker sign. With First-Person-Action, we noted that sensors that were placed along the thumb were regarded as the most discriminative ones. This is intuitive since this dataset includes activities that involve the opening and closing of bottles, cartons, and jars. For instance, opening a jar usually requires most of the surface of the thumb to be in contact with the lid. Sensors on the index and middle fingers were also indicated by the algorithm to serve as a complement for better recognition performance since these fingers are also the main ones involved in opening jar lids and bottle caps. Finally, from IPN Hand, the tips of the thumb, index, and middle fingers ranked on the top since these are the central fingers that were involved in four of the five gestures of this dataset: clicking with one finger, clicking with two fingers, zooming in, and zooming out.

Table 6 includes the classification performance results for different numbers of sensors *n* in two different types of configuration: a configuration formed by the *n* most (denoted as “B” for “best”) important sensors and a configuration in which the *n* least (denoted as “W” for “worst”) important ones were selected. Additionally, the performance with all the sensors was also included. We observed that the best selection of sensors always outperformed the worst ones and often by a considerable margin, especially for a small number of sensors. In First-Person-Action, for instance, the placement of three sensors on the thumb reached a performance considerably near having all 15 sensors on the hand. In comparison, the worst selection of three sensors for this dataset delivered an F1-score that was nearly 20% lower.

**Table 6:** A comparison of the sensor selection that was provided by our algorithm.

Dataset	# of sensors										
	All	**3**		**4**		**5**		**6**		**7**	
		B	W	B	W	B	W	B	W	B	W
InterHand^44^	0.921	0.864	0.813	0.893	0.863	0.907	0.895	0.921	0.911	0.922	0.920
First-Person-Action^45^	0.800	0.752	0.568	0.783	0.691	0.723	0.710	0.798	0.775	0.771	0.747
IPN Hand^46^	0.874	0.800	0.671	0.816	0.700	0.847	0.759	0.851	0.753	0.872	0.796

B denotes a selection that contains the most important sensors that were chosen using our algorithm, whereas W denotes a configuration with the least important sensors.

In IPN Hand, the worst selection of three sensors included locations on the index and middle fingers. However, the sensors in these locations were hardly capable of differentiating between the motions of closing the hand and zooming out. In contrast, the sensors that were placed on the tip of the index and middle fingers, as is the case of the best selection of sensors, provided enough information to differentiate between these two aforementioned motions since the tips of the index and middle fingers travel a farther distance in the gesture of closing the hand in comparison to when they zoom out.

The contrast in performance between the best and the worst selection of three sensors was lower in InterHand. From the perspective of the sensors being placed on the middle and ring fingers, the gestures of making a fist, making the rocker sign, and giving a thumbs-up looked identical since, for these fingers, the motion was the same across these gestures. However, distinctions in the orientation of the hand existed when performing the mentioned gestures. These different hand orientations were captured by the magnetometer and accelerometer, which provided enough information to perform a reasonable classification. The accelerometer contained information on orientation because the gravity component was included in the *x*, *y*, and *z* axes. As the orientation of the accelerometer changed, the decomposition of the gravity component on these axes also changed.

Still, according to Table 6, the performance disparity between the worst and best selections grew shorter as the number of sensors increased. This can be explained. A set of the best three sensors already includes a great deal of information for accurate classification. The opposite is valid for a set of the worst three sensors. Hence, going from the best three to the best four results in a smaller performance gain than when transitioning from the worst three to the worst four. The performance degradation in First-Person-Action that was witnessed when growing from the best four to the best five sensors can be explained by the erroneous simulated data that was produced by the sensor that was placed on the top of the middle finger. Such a disturbance is the cause of errors in the output of the inverse kinematics. Finally, a best selection that contains six sensors reaches a classification performance that is considerably close to a selection with all sensors.

Table 7 demonstrates the evolution of resource consumption as the number of sensors grows. Since the memory footprints and inference times almost double as the number of sensors doubles, a successful sensor placement selection—as it is the purpose of our algorithm—is crucial to resource efficiency. For instance, in IPN Hand, an F1-score of approximately 0.8 can either be achieved with three well-selected sensor locations or seven poorly selected sensors. This discrepancy of four sensors results = in a two-fold increase in resource consumption. The resource consumption report requires a Raspberry Pi 3 or 4B microcontroller from the user of the platform.

**Table 7:** The relationship between the resource utilization and the number of sensors.

Resource	Number of sensors				
	3	4	5	6	7
Memory (MB)	5.97	7.57	8.83	10.23	11.89
Inference time (ms)	17.29	20.09	22.94	28.19	32.22

The resource utilization was calculated on a Raspberry Pi 4 B device.

Since we did not exhaustively test all the possible combinations of sensor placements for all the datasets, we do not claim that our sensor selection algorithm found an optimal design. Nevertheless, the obtained placements were verified to perform significantly well, and such performances can be explained by carefully analyzing the activities.

### Case study: smart glove design

Thus far, we have evaluated the (1) inverse kinematics module that produces the workspace for the sensor simulation, (2) the data augmentation methods from which we have obtained the optimal proportions of augmented data for each dataset, and (3) the sensor selection algorithm, which has delivered a ranking of sensor locations from the most important to the least important ones. This way, we can estimate prior performance expectations for different sets of gestures and sensor placements. In the following case study, we tested a complete run-through of the whole pipeline that resulted in a smart glove design.

**Figure 12:**
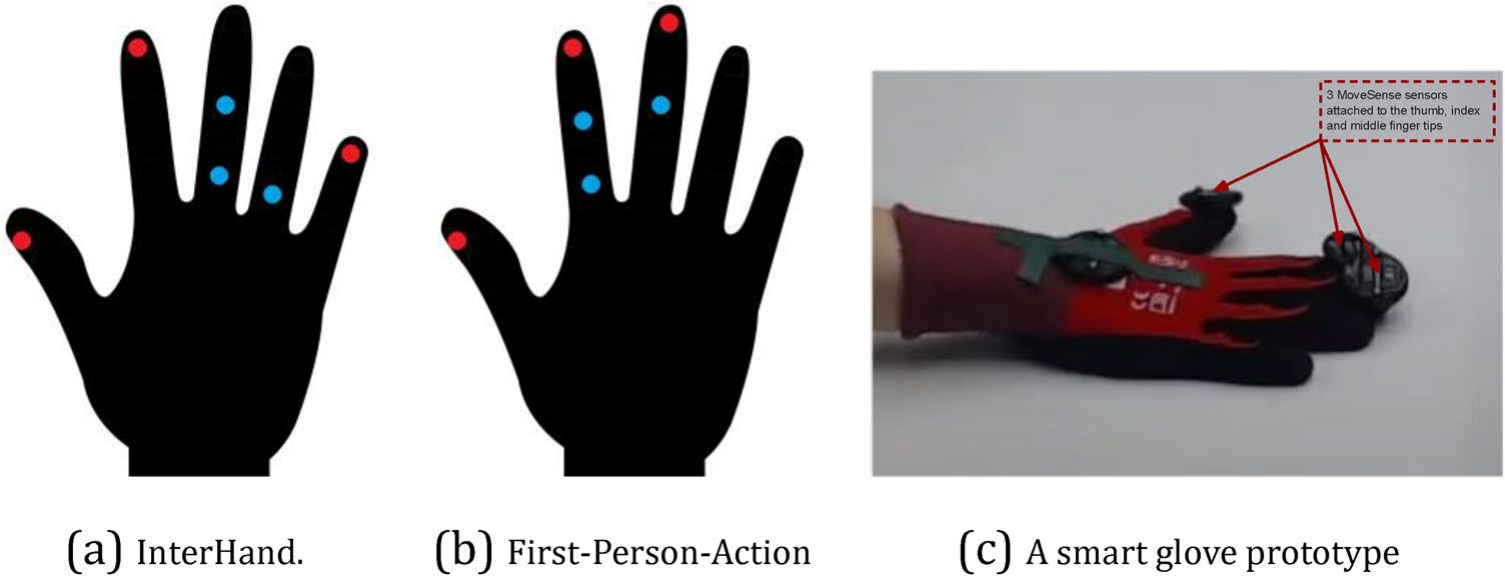
Best (in red) and worst (in blue) sensor placements that were utilized in our case study.

**Figure 13:**
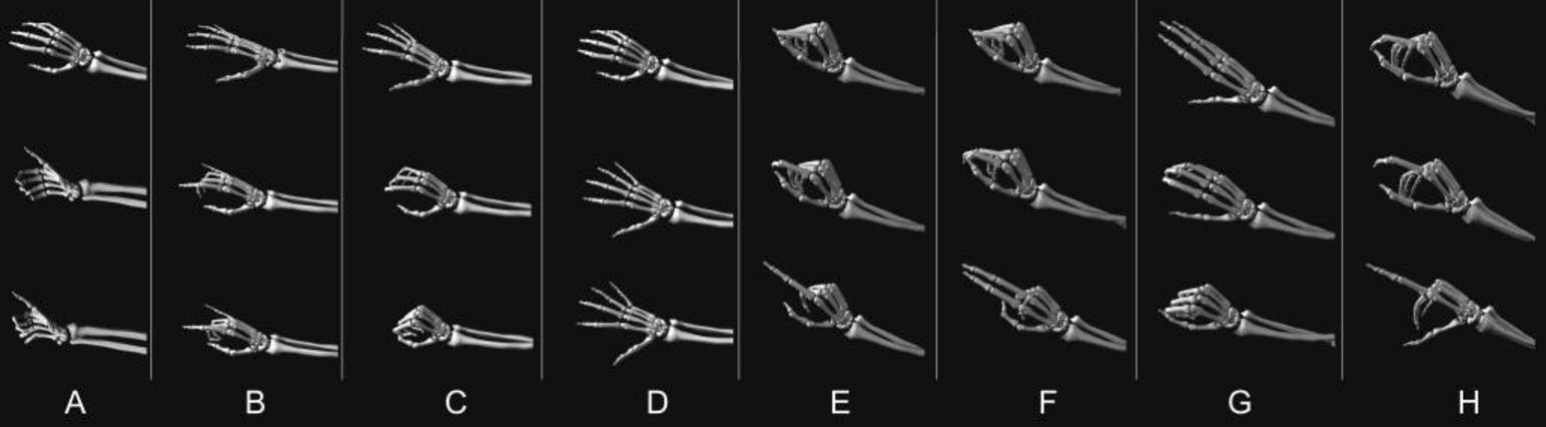
Motions that were utilized for the case study. (**A**) thumbs-up (InterHand), (**B**) rocker sign (InterHand), (**C**) fist (InterHand), (**D**) spreading fingers (InterHand), (**E**) clicking with one finger (IPN Hand), (**F**) clicking with two fingers (IPN Hand), closing hand (IPN Hand), and zooming in and out (IPN Hand).

We implemented the proposed smart glove configurations (Fig. 12c) for the datasets InterHand and IPN Hand. We utilized three Movesense sensors that were placed first according to the best sensor placement for the activities and second for the worst sensor placement for the comparison. We recorded better and worse sensor setups separately since placing more sensors limits the range of motion while performing the selected gestures. To collect real smart glove data, we selected a target subset of gestures from the InterHand dataset: giving a thumbs-up, making a fist, making the rocker sign, and spreading the fingers apart. From the IPN Hand dataset, the gesture set was formed using the activities of clicking with one finger, clicking with two fingers, closing the hand, and zooming in and out. Figure 13 illustrates the motions that were utilized in the case study. The gestures from the First-Person-Action dataset were disregarded since they included interactions with objects. The goal of the case study was to provide a full forward pass of our pipeline and showcase that it can successfully pick designs that produce the best results in a realistic scenario.

Each recording lasted for 45 s for each gesture, in which the gesture was repeatedly performed (18–30 repetitions). Every recording consisted of two sensor setup variants: the best and worst placements of the three sensors. Figure 12a and b illustrate the best (in red) and worst (in blue) configurations for each of the datasets. Finally, we utilized the augmented sensor simulated data to train a classifier that was then tested on the real data. We utilized the proportions of data augmentation that provided the optimal results in Table 4—that is, 5% and 10%of gesture style augmentation for InterHand and IPN Hand, respectively. We organized the results into three different scenarios. In *Virtual* → *Virtual*, a classifier was trained and tested on simulated data (for both best and worst configurations). The scenario *90% Virtual* + *10% Real* → *Real* consisted of a training set that was mostly composed of simulated data and a small amount of real data. This training set was used to train a classifier that was tested on real data. The last scenario, *Virtual* → *Real*, used simulated and real data on the training and test sets, respectively.

Since the exact orientation of the sensors, as well as the hand of the user, in the real data differed from that in the simulated data, during the training of the classifier, we applied a random rotation to the simulated data at each training step. Such operations were necessary to ensure that the classifier would not be biased toward one specific orientation. A multiplicative noise with a mean 1 and a standard deviation 0.2 was also applied to the simulated data since it has can improve the performance of the classifier when tested on real data, as evidenced during preliminary experiments.

#### Results

Table 8 lists the results of our case study in three different scenarios. In all cases that involved real data, the configuration of the sensors that were indicated as the best ones by our sensor selection algorithm delivered significantly better performances than the worst configurations. This can be characterized as a shred of evidence that the proposed sensor selection algorithm can be successfully applied to real-life cases. The reasonably low F1-scores that were achieved in the *Virtual* → *Real* scenario can be explained using the disparity between the real and simulated data distributions. Such performance degradation when the classifier is tested on virtual data and real data is not unique to HAR. Several other areas such as computer vision suffer from this domain shift. However, the addition of a very small portion of real data to the training set resulted in significant performance improvements. In our case study, 10% amounted to approximately 25 s of recorded data for each gesture. The results of the case study were very similar for both IPN Hand and InterHand, which indicates that using video data or motion capture data as input works well.

**Table 8:** The results of the case study experiment separated by datasets, sensor configurations, and categories of training and test sets.

Dataset	Virtual → Virtual		90% Virtual + 10% Real → Real		Virtual → Real	
	Best config	Worst config	Best config	Worst config	Best config	Worst config
IPN Hand^47^	0.892	0.669	0.753	0.604	0.438	0.341
InterHand^45^	0.888	0.813	0.759	0.684	0.617	0.381

*Virtual* → *Virtual* represents the case in which the neural network was trained on virtual data and used to predict virtual data—it represents an ideal category where the distributions of training and test data are identical. In *90% Virtual* + *10% Real* → *Real*, the neural network was trained using both virtual and real data with a proportion of 90% and 10%, respectively. Finally, the case *Virtual* → *Real* consisted of a training set that was entirely built on virtual data.

Altogether, we recommend the inclusion of small portions of real-world data in the training set. The data can be collected after our pipeline has given the best placement of sensors and simply merged together with simulated data at the final stage (i.e., the neural network training). This process can happen without any change to the pipeline.

## Discussion

### Advantages

We discuss the specific advantages of the proposed approach, which, in our opinion, distinguish it from the commonly used strategies for designing a smart glove.

#### Sensor placement optimization and data augmentation

Our pipeline performs sensor placement optimization and data augmentation directly using motion data instead of sensor data. For instance, modifying sensor data to mimic varying and realistic finger bone lengths can be an intractable task. This operation, however, can be easily executed by scaling the finger bones of a virtual hand model and simulating the sensor data from the movements of the scaled model, which is done in our pipeline. Devising a method for finding recommended sensor locations is also simpler when dealing with motion data since it is more intuitive to work with than IMU readings such as accelerations, angular velocities, and magnetic field intensities. Additionally, by using heuristics based on finger motions, our sensor placement optimization method can be more data-efficient than generic solutions.

#### Modularity

Our pipeline is modular since its components can be replaced with equivalent ones. For instance, the sensor selection and neural network architecture can be completely altered without affecting the nature of the pipeline. The data augmentation can also be modified or completely removed from the pipeline. This establishes a clear path for pipeline improvements: a technological or algorithmic innovation can be easily imported into the proposed workflow.

#### Robustness

Despite the use of noisy motion input data, which is a result of existing technical challenges regarding estimating 3D poses from monocular images, our pipeline can deliver a sensible smart glove design that can be used to solve gesture recognition in real-life scenarios.

#### Cost and time savings

The proposed pipeline provides a more cost and time-efficient approach to developing HAR since it circumvents the labor-intensive and iterative task of manual data collection and facilitates simulated prior tests and improvements of a prototype; this way, it reduces the number of intermediate prototypes. The user only needs to provide input data and choose among candidate optimal HAR designs based on their computational costs and classification performances. If a trained classifier provided by the platform is to be used, then a small portion of real-life data should be collected to improve the performance of the classifier.

It is not easy to give a concrete figure about the amount of time saved through our approach. The number of hours of work our pipeline spares (compared to the conventional design approach) heavily depends on factors as follows.

- *The number of activities.* A higher number of activities signifies longer data collection sessions for each prototype tested with the conventional design approach. That is, the benefit of utilizing our pipeline increases with the number of activities.
- *The number of subjects included in the data collection.* Collecting and annotating data is a considerably time-consuming task, especially when a high number of subjects is included in this process. Given that, in the conventional design approach, the data collection is performed several times, the accumulated duration of this task can reach weeks. Hence, the number of hours of work our pipeline can spare increases with the number of subjects for the data collection.
- *The utilization of heuristics.* In the conventional approach, an experienced HAR designer can significantly shorten the iterative process by employing diverse tactics and heuristics. Therefore, the benefits of our pipeline are higher when expert human knowledge is not available.
- *The complexity of the prototypes.* Prototypes with a larger number of sensors require a considerably longer time to be built and modified. Therefore, the benefit of our pipeline grows larger with the complexity of the prototypes.

### Limitations and future work

The primary source of noise stems from the motion data (particularly when derived from videos) and is carried throughout the entire pipeline, leading to errors in both inverse kinematics and sensor simulation. A secondary source of noise is located in the sensor simulations due to imperfect mathematical modeling of accelerometers, gyroscopes, and magnetometers. However, the impact of these errors is generally less significant compared to those originating from noise in the motion data.

Using videos with higher resolution, good lighting, and clear viewpoints can improve the output of the hand key-point extractions -– for example, in the IPN Hand dataset for approximately 15% of frames the hand key points, which were not detected due to the high pace movement which led to a blurry frame, even though those were the exact frames with high motion information that was required for the pipeline. In addition to our pre-processing transformations and filtering processes, a potential future solution involves automatically detecting unrealistic human movements within the motion data provided by the inverse kinematics module. This would allow users to quickly and easily apply corrective adjustments to the data. Another direction for enhancing the pipeline involves leveraging human pose information extracted from inertial measurements, as demonstrated by Huang et al.^48^.

The inverse kinematics module is highly sensitive to the quality of the input data. Since 3D pose estimations from monocular videos do not always follow physical joint constraints and prone to higher marker distance errors (defined in Sect. “Experiment 1: inverse kinematics”). Additionally, the key-point anchors are not consistent across different datasets—the points can be associated with the palmar side of the hand, the center of the joint rotation, or the dorsal side of the hand. These inconsistencies may also lead to higher marker distance errors. In our pipeline, we used the OpenSim musculoskeletal model of a hand with predefined mechanical properties and strict muscle constraints, which was designed to study musculoskeletal disorders. Future iterations may need a modified hand model that is more tolerant to the noise in the key-point positions.

While the implemented data augmentation is beneficial, it is limited in scope. The first part of the augmentation, which concerns hand size augmentation, covers simple hand morphology changes based on bone length statistics. This could be expanded by conducting an in-depth study of bone length relationships, which would be more representative of real human hand sizes. However, the concept of a hand is flexible. It is expected to be easier for a neural network to learn about hands from different distributions. The second part, which concerns gesture augmentation, covers variations that are close to the original motion. The scope of this study limits us to such an approach, and generating motion variants and completely synthesized motions are still open issues. Novel approaches that use generative deep neural networks represent promising steps toward solving this issue. However, they still struggle with the problem of guaranteeing the same gesture class, which is critical to our purposes.

Sensor simulation does not perfectly reproduce the real sensor readings since the mathematical model of the sensors itself and its parameters is an approximation, even if a handful of error signals are simulated, as is the case with MATLAB’s simulation. Nevertheless, the achieved simulation precision is sufficient to train on and differentiate the hand gestures reliably. Furthermore, the current pipeline focuses on the IMU sensor simulation. Some gestures and hand interactions may require different sensors for reliable recognition. In future work, we envision the addition of various sensor modalities and domain adaptation methods to bridge the gap between the simulated and real data distributions.

The results of the case demonstrated that the inclusion of a small portion of real sensory data in the training significantly improves the performance of the gesture classification model (Table 8), especially for the video-sourced motion dataset (IPN Hands). The model trained solely on simulated data does not perform satisfactorily out-of-the-box; this reaffirms the need to bridge the domain gap between real and simulated sensors. Increasing the number of used sensors may also reduce the model performance degradation when training on the simulated data. Further investigation is needed.

## Conclusions

We proposed a novel simulation platform for designing smart gloves for gesture recognition that is based on monocular videos. Our contributions in our pipeline revolve around the input data and the sensor placement optimization. Regarding the former contribution, we addressed the unreliable and inaccurate 3D pose estimations that are often extracted from monocular videos. The latter contribution consists of deriving a potential optimal configuration of sensors based on the statistics of joint angles, which are more tractable and intuitive in comparison to sensor readings such as accelerations, angular speeds, and magnetic field readings. The various stages of the pipeline were evaluated and applied to two real-life cases, from which favorable results were obtained.

## Data Availability

The datasets generated and/or analysed during the current study are available online in the following links: IPN Hand, First Person Action, and InterHand. The dataset utilized for the case study (Table 8) is available here.
